# Editorial: Rubies for ESOT!

**DOI:** 10.3389/ti.2022.10529

**Published:** 2022-04-22

**Authors:** Thierry Berney, Nuria Montserrat, Maarten Naesens, Stefan Schneeberger, Maria Irene Bellini, Thomas Neyens

**Affiliations:** ^1^ Editor-in-Chief, Transplant International; ^2^ Deputy Editor-in-Chief, Transplant International; ^3^ Social Media Editor, Transplant International; ^4^ Statistical Editor, Transplant International

Forty years ago, on April 28th, 1982, ESOT was founded in Zürich, Switzerland by an assembly of 14 European transplant surgeons, to satisfy the “need for a society … which would represent the aims and needs of transplantation surgery and surgeons in Europe” (1). The founders had the wisdom to suggest that all scholars actively involved in organ transplantation should be included in such an organization rather than transplant surgeons only. This idea prevailed thanks to the visionary Sir Roy Calne who would become ESOT’s first president (1). Thus, instead of a European Society of Transplant Surgeons (ESTS), as originally planned, the European Society for Organ Transplantation (ESOT) was born. While surgeons were driving (and dominating) the world of transplantation in the 1980’s, Sir Roy had understood the need to build a broader European transplant community inclusive of physicians and scientists, meeting in a common forum, rather than at parallel events. This vision has endured and ESOT progressively engaged other categories of transplant professionals: transplant coordinators, allied healthcare professionals, and - more recently - patients and (bio)technology scientists. This last advancement is the response to the broadening of our field towards organ reconditioning, regenerative medicine, artificial and bioartificial organs (2).

The founding assembly of ESOT was a group of formidable individuals including Guy Alexandre, Max Dubernard, Carl Groth, Walter Land or Raimund Margreiter, to name only a few. One of these founding fathers was Gauke Kootstra, transplant surgeon in Maastricht, the Netherlands. Gauke had pioneered the development of machine perfusion for marginal organs (3), but his claim to fame has been the understanding of the value of “non heart-beating donors” for increasing the donor organ pool (4). His efforts contributed significantly to the standardization and categorization of donors with circulatory death (DCD), which led to the Maastricht classification (5).

The ESOT founders understood the need for the society to establish a scientific journal and Gauke Kootstra was appointed as the first editor-in-chief of *Transplant International* (1988–1998). As a result of the strong commitment of the ESOT community, the editorial board released the first issue containing 10 articles in 1988 (6). Since then, *Transplant International* has successfully established itself as a major and respected title and has consistently progressed under the stewardship of Ferdinand Mühlbacher (1999–2014) and the team of Thomas Wekerle and Rainer Oberbauer (2015–2021) in their roles as editors-in-chief. The current leading editorial board is grateful to have inherited such a high-quality journal from our forebears.

From its creation, *Transplant International* was committed to modernity. As written in the editorial of the first issue: “We are convinced that with such modern means of communication as the fax, it is possible to keep the processing and publication times in our journal to a minimum. This will be of great importance, especially in the field of transplantation which is, by its nature, a very dynamic science” (6). Thirty-four years later, the fax looks like a prehistoric communication tool, but the spirit of the current editorial board is pretty much the same, embracing all the tools brought by the information revolution, for the communication with our readers and ESOT members and for the dissemination of our authors’ work (7). This has allowed to strengthen further the ties between ESOT and *Transplant International* and to work toward our common interests: the desire to address the new cutting-edge topics developing fast in the field of organ replacement (2, 8, 9). Anticipating, rather than adjusting to the modernization of scientific publication led us to adopt a gold open access model (10). The immediate -and huge-benefit is already palpable with the free access granted to all present and past *Transplant International* publications.

Traditionally, 40 years mark a ruby anniversary. As *Transplant International* has elected to go for gold, we offer (virtual) rubies to ESOT!
